# The Potential Role of Hydrogen Sulfide in the Regulation of Cerebrovascular Tone

**DOI:** 10.3390/biom10121685

**Published:** 2020-12-16

**Authors:** Eleni Dongó, Levente Kiss

**Affiliations:** 1 Department of Physiology, Semmelweis University, 1088 Budapest, Hungary; 2 Department of Neurology, Semmelweis University, 1088 Budapest, Hungary; dongo.eleni@med.semmelweis-univ.hu

**Keywords:** hydrogen sulfide, cerebral circulation, gasotransmitters

## Abstract

A better understanding of the regulation of cerebrovascular circulation is of great importance because stroke and other cerebrovascular diseases represent a major concern in healthcare leading to millions of deaths yearly. The circulation of the central nervous system is regulated in a highly complex manner involving many local factors and hydrogen sulfide (H_2_S) is emerging as one such possible factor. Several lines of evidence support that H_2_S takes part in the regulation of vascular tone. Examinations using either exogenous treatment with H_2_S donor molecules or alterations to the enzymes that are endogenously producing this molecule revealed numerous important findings about its physiological and pathophysiological role. The great majority of these studies were performed on vessel segments derived from the systemic circulation but there are important observations made using cerebral vessels as well. The findings of these experimental works indicate that H_2_S is having a complex, pleiotropic effect on the vascular wall not only in the systemic circulation but in the cerebrovascular region as well. In this review, we summarize the most important experimental findings related to the potential role of H_2_S in the cerebral circulation.

## 1. Introduction

Cases of ischemic and hemorrhagic stroke represent leading causes of death as these are responsible for nearly 6 million deaths/year globally [1]. The major effect of these diseases on the healthcare system and on the community is also underlined by the number of chronic disability cases that are caused by them [2]. Subarachnoideal bleeding is another severe condition where not only the bleeding itself but the consequential vasospasm constitutes a problem [3]. In addition to these life-threatening diseases a division of primary headaches (cluster headache, migraine) are also accompanied with vascular malfunction [4,5]. Thus, a large number of cerebrovascular diseases [6] underline the importance of better understanding of cerebrovascular regulation and achievements in this field may have great clinical impact.

## 2. Factors Regulating Cerebrovascular Tone

The circulation of the central nervous system is regulated in a complex manner and it is mainly controlled by local factors while the neural control, that is so critical in the systemic circulation, is relatively weak and even controversial in the cerebral circulation [7,8,9]. The local factors involve several mechanisms such as myogenic, flow or shear mediated and metabolic responses which were concisely reviewed recently [10] and only a brief summary is provided here. It is important to emphasize that the autoregulation of blood flow in the intracranial space is of great importance, and this is thought to be the result of a multi-factorial complex mechanism which involves myogenic response, shear stress, hyper- and hypocapnia and hypoxia as well [9,11,12]. The range of autoregulation in physiological circumstances is between 50 mmHg and 150 mmHg mean arterial pressure in the systemic circulation [9]. The coupling of blood flow to metabolism provides the necessary local changes in brain circulation for the actual neuronal activity. Rising local CO_2_ level efficiently increases cerebral blood flow through vasodilation, while lowered PaCO_2_ decreases blood flow through increased arterial resistance via the alteration of perivascular pH [13]. Vasoactive substances like H^+^ and K^+^ are also released in case of low cerebral blood flow causing vasodilation [9,13]. Neurotransmitters and neuromodulators (such as acethylcholine, catecholamines, neuropeptides) induce Ca^2+^-waves in the adjacent astrocytes and neuronal dendrites resulting in the release of further vasoactive molecules such as nitric oxide (NO, the formerly known EDRF) and/or arachidonic acid metabolites [9,13,14,15].

### Gasotransmitters in Cerebral Circulation

The first identified gasotransmitter molecule in the mammalian circulation was NO [16] which was shown to increase cerebral blood flow by decreasing vascular resistance in the cerebral vessels similarly to its effects in the systemic circulation [17]. NO in the central nervous system may originate from endothelial cells (produced by endothelial NOS, eNOS), from autonomic nitrergic nerve endings or from neurons of the central nervous system (produced by neuronal nitric oxide synthase, nNOS), or by the inducible isoform of the enzyme (iNOS) [17,18]. Decreased availability of NO is known to be a triggering factor of vasospasm after subarachnoid hemorrhage, whereas overproduction through iNOS occurs during inflammation or cerebral ischemia [18]. Several evidences provided by experiments involving animals indicate that the NOS enzyme contributes to the neurovascular coupling (known as functional hyperemia) and a recent study demonstrated this in humans as well [15,19]. However, it is still under debate if NO had a role in hypercapnia-induced vasodilation [17].

Subsequently, it was realized that not only NO but other endogenously produced molecules such as carbon monoxide (CO) can also play a role in the regulation of cerebrovascular system [20]. Endogenously produced CO originates from the metalloprotein heme by its synthesizing enzyme, heme oxygenase (HO), which has two distinct isoforms, the inducible HO-1 and the constitutive HO-2 [18,21]. The production of CO in the central nervous system takes place in the cerebral vascular cells, neurons and glial cells by HO-2, which has the highest concentration in the brain [22]. CO in the central nervous system influences neuronal activity, affects local regulation of cerebral circulation and supports antioxidant defense [22]. The vasodilatative effect of CO was proven by several studies [21,23,24]. The mechanism behind the vasodilation includes the activation of K_ATP_-channels and the cGMP pathway seems to be also affected [18]. Furthermore, CO-induced vasodilation occurs through activation of K_Ca_ channels, and HO is also presumably involved in hypoxic cerebrovascular dilatation [21,23,24,25]. It should be also noted that some studies reported vasoconstriction after CO treatment, probably through the inhibition of NOS [22]. According to these results, CO derived from HO-2 may serve as a tonic vasoregulator antagonizing NO-mediated vasodilation in the rat cerebral microcirculation [26]. Furthermore, through elevation of cGMP levels, NO can inhibit directly and activate indirectly the HO-2 isoform [22]. Taken into account these interactions, a quite complex feedback control is probable [22].

Importantly, among the many small molecules (such as hydrogen peroxide and potassium ion) that were implicated as potentially having important roles in the vasoactivity of cerebral vessels, hydrogen sulfide, the “third gasotransmitter” is also present [18,27,28,29,30].

## 3. Hydrogen Sulfide as the Third Gasotransmitter

There is emerging knowledge about H_2_S as an endogenously produced molecule with several effects in the living organisms, therefore it can be considered as the third gasotransmitter. H_2_S is a water-soluble, colorless, flammable gas and a weak acid which is mainly dissociated (approx. in 80%) to hydrosulfide anions (HS^−^) and hydrogen ions (H^+^) at physiological pH and body temperature [31]. Four enzymatic pathways are responsible for the enzymatic production of hydrogen sulfide in the human body. In the cardiovascular system, cystathionine-gamma-lyase (CSE) produces the majority of endogenous H_2_S which has a role in the regulation of vascular tone and angiogenesis and may have cardioprotective potential as well [32]. In the nervous system, cystathionine beta-synthase (CBS) is the main H**_2_**S producing enzyme and it is localized in astrocytes but CBS was detected in neuronal stem cells as well [33,34]. Roles of CBS could potentially involve protection of the endothelial function by anti-inflammatory, antioxidant and pro-angiogenic effects [35]. Both enzymes use pyridoxal-5-phosphate (PLP) as a cofactor [36]. 3-mercaptopyruvate-sulfurtransferase (MST) and cysteine amino-transferase (CAT) represent the third enzymatic pathway producing H_2_S in various cell types (endothelial cell, vascular smooth muscle cells, cerebellar Purkinje-cells) [34]. A recently discovered fourth enzyme, the D-amino acid oxidase (DAO), uses D-cysteine to produce H_2_S and this pathway occurs mainly in the kidney and in the cerebellum [37]. Furthermore, non-enzymatic processes that produce H_2_S from elementar sulfur can be found in erythrocytes and gut bacteria [31]. However, it is important to note that although the details of endogenous H_2_S production are fairly well-known, the precise measurement of the H_2_S levels in biological samples is still a challenge, due to the volatile character of H_2_S and its extensive interactions with other compounds [38].

As a result of its widespread effects under physiological and pathophysiological circumstances, the examinations regarding the actions of H_2_S on the vascular system are of great interest [39,40,41,42,43,44]. Based on multiple studies the three main mechanisms behind the biological effects of H_2_S are: (1) persulfidation/polysulfide generation, (2) interaction with reactive oxygen and nitrogen species and (3) reaction with metalloproteins [36]. Persulfidation is a post-translational modification where a thiol group is added to a specific cysteine residue of a protein (enzymes, ion channels, etc.), resulting in increased or blocked biological activity [36,45]. We have concisely reviewed the possible consequences of these mechanisms on the regulation of the vascular tone in our recent review [36].

Considering the interactions of H_2_S with other gasotransmitters many such possibilities can be mentioned. H_2_S and NO has synergistic effect on the vasodilation of rat pial arteries. The effect of H_2_S seems to be more pronounced in smaller (diameter being less than 20 µM) arteries while the NO-mediated vasodilation has bigger role in larger arteries [46]. H_2_S induces vasodilation through interaction with the NO-sGC-cGMP-PKG pathway [40,46]. There are several major H_2_S-NO common products as well such as nitrosopersulfide (SSNO−), polysulfides and dinitrososulfite [N-nitrosohydroxylamine-N-sulfonate (SULFI/NO)] each with distinct biological effects [47]. One of the best known NO-H_2_S common molecule, nitroxyl (HNO), is formed from SULFI/NO and is proven to have its own effect on the vascular tone [47]. Additionally, the H_2_S-NO crosstalk in meningeal arteries induces vasodilation through the activation of the HNO-TRPA1-CGRP pathway [4]. Considering another gasotransmitter, studies in mice revealed that CO produced from HO-2 constitutively inhibits CBS in normoxic circumstances, but in the case of hypoxia, CO level drops and CBS is able to generate H_2_S finally resulting in vasodilation [48]. According to the results, this interaction between CO and H_2_S is supposed to be relevant under physiological conditions [48]. In this context, it is noteworthy that in another study (made on newborn pigs) CSE did not play a role in hypoxic vasodilation [49].

## 4. Experiments on the Involvement of H_2_S in the Cerebrovascular System

There are several possible avenues in investigating the vasoactive functions of H_2_S in the cerebrovascular system. Regarding the used methods the majority of research on cerebral circulation is done by wire myography [50,51,52,53] pressure myography [54,55] or both [56]. Patch-clamp techniques are also frequently used [54,55,56]. A peculiar approach, the surgically created cranial window allows repeated in vivo examinations in the same animal [49] while histological studies and stainings help to identify the characteristic enzyme type of a distinct tissue sample [3]. Regarding the concepts of the studies one possible and widely used option is to examine the effects at a higher H_2_S level than the endogenously produced amount. In this case, the following two methods are used the most often: (1) administration of L-cysteine, which is the universal substrate of the producing enzymes; (2) administration of pure H_2_S gas or H_2_S donor molecules. Considering the method of H**_2_**S-administration several possible routes are used in experiments involving animals. In vivo studies are using many times intraperitoneal [3,57,58] administration or—in cerebrovascular studies—intraventricular injection [58] and even topical treatment through a previously prepared cranial window [46]. In human studies involving cerebrovascular questions, exogenous H**_2_**S treatment was not performed, only serum cysteine level was measured in a study including post-stroke patients [58]. However, intravenous administration was performed in certain studies exploring the role of H**_2_**S on systemic circulation [47,59].

Experiments and their major conclusions using H_2_S donor treatments in in vitro, ex vivo and in vivo settings are summarized in Table 1, Table 2 and Table 3, respectively.

Another possibility in the investigation of the possible roles of hydrogen sulfide in living systems is the inhibition of the enzymes that are producing it. This method may also help to elucidate which enzyme is responsible for the H_2_S production in a given tissue. Such experiments are summarized in Table 4.

There are further possible approaches which are not falling into the abovementioned two categories. Some of these are focusing on the importance of endogenous H_2_S-production in animals that lack one of the synthesizing enzymes (CSE^−/−^ or CBS^−/−^) while others are using disease models or other vasoactive agents and focus on the plasma or tissue concentrations of hydrogen sulfide (Table 5).

We summarize here some of the key messages from the tables and we also provide a versatile Excel table as a supplement to this article in which individual considerations can be taken into account and rearrangements of the publications can be made accordingly (Supplementary Table S1).

In pig pial arteries and rat middle cerebral arteries, the CSE-inhibitor propargylglycine (PAG) was proven to be effective in counteracting the vasodilator effects of H_2_S, while the aminooxyacetate (AOAA), which is used as CBS inhibitor, had no remarkable effect [49,52,56]. Therefore, the cerebral vasculature probably produces H_2_S mainly by CSE. However, in neonatal mice cerebellar slices the constitutively produced CO inhibited the CBS dependent H_2_S generation, and in hypoxic conditions (modeled by treatment with the HO inhibitor ZnPP) the lowered CO level enabled increased H_2_S production leading to vasodilation in which K_ATP_ channel activation played a role [48]. Still, the vasorelaxant effect of NaHS was more pronounced in CBS^−/−^ mice compared to WT animals which indicates that a supersensitivity to H_2_S is present after the depletion of endogenous H_2_S [48]. In an experiment that involved pressure myograph examinations on rat cerebral arteries exogenous NaHS induced relaxation of the vessel segments [56]. Furthermore, other studies have also proved that NaHS induced vasorelaxation at higher concentrations on rat middle cerebral arteries [51,52] and on basilar artery segments derived from mice as well [53]. NaHS induced dose-dependent relaxation in rat middle cerebral arteries, which was reduced by nifedipine (voltage-gated Ca^2+^-channel blocker) and inhibited by 50 mM K^+^ also, but selective blockers of K_ATP_, K_Ca_, K_V_ or K_ir_ channels failed to repeat this effect [51]. However, in a study on mouse cerebral arterioles, the vasodilator effect of H_2_S was absent in SUR2-null animals, supporting the role of K_ATP_ channels in this species [54]. Although treatment with the non-specific Cl^−^ channel and anion exchanger inhibitor 4,4’-Diisothiocyano-2,2’-stilbenedisulfonic acid (DIDS) caused a rightward shift in the NaHS concentration-response curve, results after using more selective blockers of Cl^−^ channels, such as niflumic acid, and results with the use of HCO_3_^−^ free solution question the involvement of Cl^−^/HCO_3_^−^ exchange in the effects of H_2_S. Thus, DIDS, which is known to be non-specific in its actions, probably alters some other action of NaHS [51]. In this study, the vasodilation caused by NaHS did not depend on the presence of intact endothel; therefore, H_2_S was supposed to be mainly produced in the vascular smooth muscle layer [51].

It might be important to separately consider the several investigations that were focusing on the effects of H_2_S in pigs. In newborn pigs, H_2_S produced by CSE appeared to be a functionally significant vasodilator molecule on pial arterioles, and it also had a role in the hypercapnia-induced vasodilation [49]. Opening of the K_ATP_ channels were found to be involved in the effect of NaHS in pig cerebral circulation [49,54]. Considering experiments that involved pressure myograph examinations on piglet cerebral arterioles, one can conclude that exogenous NaHS induced relaxation of these vessel segments [54,55]. In newborn pig pial arteries lower concentrations of endothelin-1 (ET-1) had vasodilatory effect through CSE/H_2_S while a vasoconstrictor effect occurred at higher concentrations and it was independent from the activity of endogenous H_2_S synthesis [60]. Therefore, it is important to note that vasoconstrictor effects of H_2_S were also described in the cerebrovascular circulation similarly to the systemic circulation [61]. In a study on rat basilar artery segments, NaHS induced vasoconstriction between 10^−7^ and 10^−3.5^ M concentrations. Interestingly, the vasoconstrictor effect weakened at 10-3.5 M and NaHS had the greatest vasoconstrictor potential while Na_2_S induced lesser constriction, while the slow-releasing donor GYY4137 had the minimal effect [50]. This NaHS induced constriction on basilar artery segments was enhanced by isoprenaline and forskolin therefore suggesting that H_2_S exerted its effects through the cAMP/adenylyl cyclase pathway [50]. Moreover, this vasoconstriction was thought to take part in setting the normal vascular tone on the basilar arteries by counteracting the vasorelaxation by NO [50]. In the same study on human brain vascular smooth muscle cells (HBVSMCs), NaHS attenuated the increase of cAMP levels after isoprenaline treatment and the enhanced adenylyl cyclase activity after forskolin, strengthening further that H_2_S acted through the modulation of the β-adrenergic receptor related pathways [50].

In addition to the abovementioned studies that were investigating the effects of hydrogen sulfide in models that do not involve disease conditions, there were several ones looking into the role of H_2_S in the pathogenesis of cerebrovascular diseases. H_2_S showed beneficial effects in a rat model of middle cerebral artery occlusion as hydrogen sulfide promoted angiogenesis, endothelial cell synthesis and migration, and also exerted neuroprotective effects after ischemia [57]. In a rat model of subarachnoid hemorrhage, NaHS lowered the vascular tone in cerebral vasospasm [3]. According to a study on rat pial arteries, after cerebral ischemia-reperfusion, the NO-mediated vasodilation is affected more severely than the H_2_S-induced mechanisms, therefore this latter pathway has important role under these circumstances [46]. Examination of acute stroke patients revealed positive correlation between the serum cysteine levels and the clinical outcome of stroke [58]. However, in rat middle cerebral arteries, administration of cysteine increased the infarct volume, and this effect was inhibited by aminooxyacetic acid (AOAA, a CBS inhibitor) [58]. Therefore, it is still under debate whether post-stroke exogenous hydrogen sulfide therapy would exert beneficial effects or if it would even aggravate the tissue damage.

## 5. Potential Roles of H_2_S in the Context of Cerebrovascular Tone

Analyzing the literature presented in the tables, one can note that the cerebrovascular effect of H_2_S that most investigations are indicating is a vasodilatory response. It is of interest though that the precontraction levels are very varied among the experiments and in some cases the H_2_S related effects are even measured on the spontaneous tone of the used vessel [51,52]. Therefore, the concentration ranges where H_2_S may act as a vasodilatory molecule are ranging from as low as 10 µM to 1 mM. In this context the used H_2_S donor is probably very important as well. Within the group of H_2_S donors, fast- and slow-release compounds can be distinguished. Fast releasing donors are sulfide salts, like sodium hydrosulfide (NaHS) or sodium disulfide (Na_2_S) [62] and an important limitation regarding their use can be derived from their name. These donors lead to extremely high levels of H_2_S immediately after their administration and then the level decreases rapidly resulting in highly fluctuating H_2_S concentrations [62]. The slow-releasing donors (GYY4137, and its further derivatives) provide a more reliable dosage and a more sustained level of H_2_S during treatment [63,64]. Interestingly, only one study about the cerebrovascular effect of H_2_S included measurements with GYY4137 and according to the results of this publication, GYY4137 had the smallest effect on vascular tone compared to the fast-releasing compounds NaHS and Na_2_S [50]. It was suspected that the difference might relate to the abovementioned H_2_S-releasing properties of GYY4137 [50].

**Figure 1:**
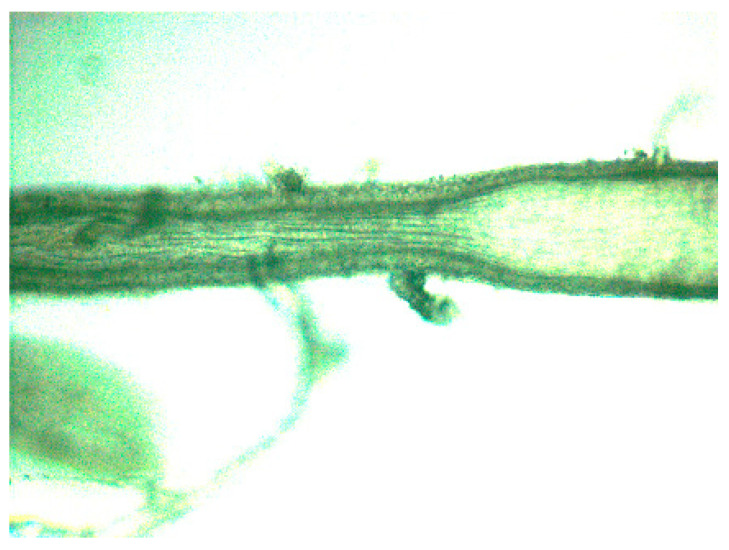
Effect of 1 mM of NaHS on rat anterior cerebral artery segment precontracted with 3 µM U46619 on a pressure myograph setup at 50 mmHg. The side branch in the middle was leaking and therefore flow occurred in the left part of the segment while no flow was present on the right which led to separate effects, namely, vasoconstriction where flow was present and vasodilation where no flow occurred.

The vasodilatory response involves similar mechanisms to those main effects that are reported in the systemic circulation, namely, interference with potassium channels with the NO-pathway and with voltage dependent calcium channels [36]. In this context, it is important to realize that interstitial K^+^ levels may be more variable in the cerebrovascular region than in the systemic circulation due to the neuronal action potential firing related K^+^ efflux. As many types of potassium channels are thought to be involved in the effects of H_2_S, this difference is probably important when we consider the net effects of H_2_S in the cerebrovascular system. It is also noteworthy that instead of the periadventitial adipose tissue [65] astrocytic endfeets are found here and a very tightly regulated neurovascular coupling is present. In some experiments, vasoconstriction was also indicated [50] and this could be potentially very important as the use of H_2_S was implied in the therapy of stroke [66]. The mechanism of action here might be related to interference with NO production [61] or decreasing cAMP levels in smooth muscle cells [50]. As shear stress is important in physiological NO production, the potential vasoconctrictor effect of H_2_S might be flow-dependent; therefore, complete understanding is not entirely possible with the most widely used wire myography methods. This might be a reason for the relatively few publications on the vasoconstrictor effects of H_2_S. That the flow and the vasoconstriction may be interrelated in the context of H_2_S is supported by our yet unpublished observations as well where we detected vasoconstriction to 1 mM of NaHS in vessel areas where flow was present while observing vasodilation where no flow occurred (Figure 1). This was achieved by an unplanned constellation during our pressure myography measurements in which constant pressure was usually generated by a pump that pushed our Krebs solution into the vessel only from the left side and the right side was closed down. In normal circumstances, this led to a constant pressure of 50 mmHg in the vessel segment without flow. However, when a supposedly ligated side branch remained open in between the cannulated rat anterior cerebral artery segment, flow occurred in one part of our segment but no flow on the other side of the leaking side branch.

Based on the analyzed experiments and the abovementioned considerations and reconsidering the local factors involved in the cerebrovascular tone (myogenic, flow or shear mediated and metabolic responses), it seems probable that H_2_S may have a role mostly in the metabolic regulation especially in the coupling of oxygen supply to the needs of neurons either via CO dependent CBS inhibition [48] or via CO_2_ dependent pH changes that influence CSE activity [49]. Interestingly, H_2_S was already implicated in both oxygen dependent vasoconstriction and vasodilation in other circulatory regions [67]. Still, the complex interactions with the NO system [47] are far from being understood, especially in the context of the cerebral circulation, therefore H_2_S may have an influence on the flow mediated regulation as well, which is supported by our own abovementioned observation.

## 6. Conclusions

Hydrogen sulfide definitely has the capability to alter the tone of cerebrovascular smooth muscle and its effects are highly dependent on such circumstances that are highly variable in the cerebral circulation. The actual myogenic tone, the flow mediated production of NO, the actual PaCO_2_ and PaO_2_ levels are all involved in a complex equation regarding the net effect of H_2_S. The pleiotropic nature of its mechanisms of actions and their dose- and time-dependency further complicate the situation. Deciphering this equation is highly difficult and certainly necessitates further studies with more and more refined experimental tools but the clinical importance of cerebrovascular diseases definitely warrants such efforts.

## Figures and Tables

**Table 1:** In vitro investigations involving H_2_S donor treatment.

Study Type	Experimental Situations	Major Conclusions	Ref
Cell culture studies on mouse brain endothelial cells (MBECs) and type I. clone astrocytes	5 µM NaHS treatment for 0-20-60-180 min during oxygen-glucose deprivation, or incubation of MBECs with 5 µM NaHS and the PI3K inhibitor LY294002 in a wound healing assay	NaHS treatment increased VEGF, angiotensin-1 (Ang-1) expression during oxygen-glucose deprivation and also increased endothelial cell migration and tube formation	[57]
Study on Sprague-Dawley rats with Western blot and immunofluorescent staining and TUNEL assay on brain sections	Middle cerebral artery occlusion with 120 min ischemia followed by reperfusion. 5 mg/kg ip. NaHS treatment from the 3rd to the 14th day after reperfusion.	NaHS treatment resulted in augmented angiogenesis in the peri-infarct area; improved functional outcomes; phosphorylation of AKT and ERK increased; VEGF, Ang-1 expression also increased	[57]
Patch clamp study on single myocytes from isolated rat cerebral artery	Effects of 1–10 mM NaHS on L-type Ca^2+^-currents	NaHS dose-dependently inhibited L-type Ca^2+^-currents	[56]
Patch clamp study on isolated arteriole derived smooth muscle cells from newborn piglets	Effects of 10 µM Na_2_S on K_Ca_ currents	H_2_S increased K_Ca_ current frequency, but did not affect the amplitude; the treatment had no effect directly on the K_Ca_ channels but only on Ca^2+^ sparks	[55]
Patch clamp study on cells from pial arteries from newborn piglets	Measurement of K^+^-currents after pinacidil, 10 µM Na_2_S or 20 µM NaHS treatment, and studying the effect of glibenclamide (K_ATP_ inhibitor)	Pinacidil, Na_2_S and NaHS induced K^+^-currents through K_ATP_ channels. Glibenclamide fully reversed the effect of pinacidil, and partially reversed the effect of H_2_S-donors	[54]
Study on basilar arteries after experimental subarachnoid hemorrhage (SAH) in Wistar rats	Measurement of CBS and CSE expression after 0.18 mmol/kg NaHS ip. treatment	NaHS increased CBS expression in both the control and SAH groups; CSE expression did not change between groups	[3]
Study with cAMP level immunoassay and adenylyl cyclase activity assay on human brain vascular smooth muscle cells (HBVSMCs)	Measurement of cAMP level after 10^−4^ M NaHS treatment, isoprenaline and forskolin treatment	NaHS attenuated the elevated cAMP levels after isoprenaline treatment; also attenuated the enhanced adenylyl cyclase activity after forskolin	[50]

**Table 2:** Ex vivo investigations involving H_2_S donor treatment.

Study Type	Experimental Situations	Major Conclusions	Ref
Wire myograph study on basilar artery segments from Sprague-Dawley rats	Effects of 10^−7^–10^−3.5^ M NaHS,Na_2_S, GYY4137 on basilar artery segments	NaHS induced dose-dependent vasoconstriction; the effect decreased at 10^−3.5^ M concentration. NaHS had the highest contractile potency, followed by Na_2_S and GYY4137 had a low contractile potency	[50]
	effects of NaHS, given at the same time/after isoprenaline or forskolin treatment	If isoprenaline or forskolin was given after NaHS the vasocontriction was augmented. When NaHS and isoprenaline/forskolin were given together, the vasorelaxant effect of isoprenaline decreased.	
	effects of NaHS given with 8B-cAMP (analog of cAMP) and Bay K-8644 (selective L-type Ca^2+^-channel blocker)	In the presence of 8B-cAMP, the vasocontriction was diminished; Bay K-8644 had no effect	
	NaHS treatment on endothelium-denuded segments or in the presence of L-NAME	Endothelium removal or L-NAME enhanced the NaHS-induced vasoconstriction	
Wire myograph study on middle cerebral artery segments of Sprague–Dawley rats	Effects of 10 mM L-cysteine on the vascular tone;	L-cysteine dilated the artery segments	[56]
	Effects of 0.5-1-10 mM NaHS on CaCl_2_-induced contraction	NaHS inhibited the CaCl_2_-induced contractions	
	0.03–10 mM NaHS treatment with K^+^-channel blockers (glibenclamide, TEA, 4-aminopyridine, BaCl_2_), with nifedipine, L-NAME or with indomethacin	NaHS caused dose-dependent relaxation, which was unaffected by the K^+^-channel blockers, L-NAME or indomethacin, but its effect was decreased by nifedipine	
Wire myograph study on mouse basilar arteries	Effects of 10^−6^–10^−3^ M NaHS on vascular tone given with LY27632 ROCK inhibitor	H_2_S induced concentration-dependent relaxation; it was attenuated by LY27632	[53]
Wire myograph study on middle cerebral artery segments of Sprague–Dawley rats (WT and STZ-induced diabetic)	Measurements after 10 µM-100 mM L-cysteine treatment	L-cysteine induced vasorelaxation in both groups, but the effect was enhanced in the diabetic group	[52]
	Measurements with 10 µM-3 mM NaHS using KCl, DIDS, nifedipine	NaHS induced equal vasorelaxation both in the diabetic and the normal group; the contribution of Cl^−^ channels were less in the diabetic group	
Wire myograph study on middle cerebral artery segments from Sprague–Dawley rats	Measurements with 100 µM-3 mM NaHS on intact and on endothelium-denuded vessel segments treated with nifedipine, K_ATP_, K_Ca_, K_V_, K_ir_ blockers	NaHS induced concentration-dependent relaxation unaffected by endothelium removal; nifedipine and high [K^+^]_EC_ decreased the relaxing effect, but blockers of K_ATP_, K_Ca_, K_V_, K_ir_ channels had no effect.	[51]
	Measurements with 100 µM-3 mM NaHS using DIDS	DIDS caused significant rightward shift in the concentration-response curve; but not by inhibition of Cl^−^ channels or Cl^−^/HCO_3_^−^ exchange	
Pressure myograph study and confocal imaging on middle cerebral artery segments from Sprague–Dawley rats	Effects of 100 µM–10 mM NaHS on myogenic tone	NaHS reduced myogenic tone	[56]
	Effects of 0.1-0.5-1 mM NaHS on [Ca^2+^]_i_ level	NaHS decreased [Ca^2+^]_i_	
Pressure myograph study and confocal imaging on cerebral arterioles from newborn piglets	Effects of iberiotoxin on 10 µM Na_2_S induced hyperpolarization;	H_2_S induces iberiotoxin-sensitive hyperpolarization	[55]
	Effects of 10 µM Na_2_S alone/with iberiotoxin or ryanodine	H_2_S induced vasodilation; it was partially reversed by iberiotoxine and also partially reversed by ryanodine	
	Effects of 10 µM Na_2_S on Ca^2+^ signals	H_2_S increased Ca^2+^-spark frequency, decreased [Ca^2+^]_IC_ levels and increased caffeine-induced [Ca^2+^ ]_IC_ transients; elevated [Ca^2+^]_SR_ transients	
Pressure myograph study on pial arterioles of newborn piglets	Measurements of vascular response to 0.1–1000 µM Na_2_S and after glibenclamide	H_2_S induced vasodilation, which was attenuated by glibenclamide	[54]
Pressure myograph study on posterior cerebral and cerebellar arteries of wild-type and SUR2-null mice	Measurements of vascular response to 5 µM Na_2_S and pinacidil in WT and SUR2-null animals	Na_2_S- and pinacidil-induced vasodilation was attenuated in the SUR2-null mice	[54]
Study on basilar arteries after subarachnoid hemorrhage (SAH) in Wistar rats	Effects of 0.18 mmol/kg NaHS ip. on luminal diameter and wall thickness	NaHS had a vasodilatory effect, the luminal diameter increased both in the control and in the SAH group while wall thickness was decreased in both groups.	[3]
Study on wild type (WT) and CBS-knock out (CBS-KO) mouse vermis arterioles from cerebellar slices	Treatment on the cerebellar slices either with the HO-inhibitor ZnPP or 30 µM NaHS; studying the effect of glibenclamide treatment	In WT mice ZnPP elicited vasorelaxation through blockade of CO comparably to the effect of NaHS. In CBS-KO mice, ZnPP treatment did not result in vasodilation; the effect of NaHS was more pronounced, but it was abolished by glibenclamide	[48]

**Table 3:** In vivo investigations involving H_2_S donor treatment.

Study Type	Experimental Situations	Major Conclusions	Ref
Study on pial arteries using cranial windows on Wistar rats	Effect of 30 µM NaHS on the vascular tone given with SNP or glibenclamide	NaHS induced vasodilation; NaHS and SNP produced greater vasodilation together than alone; inhibition of NO production or administration of glibenclamide decreased the vasodilatory effect of NaHS	[46]
Study on meningeal arteries using cranial windows on Wistar rats	Effects of 300 µM Na_2_S on the meningeal blood flow; Examinations on the role of the CGRP pathway	Na_2_S caused rapid increase in the meningeal blood flow and in the CGRP release; CGRP receptor or TRPA1 channel antagonists abolished the effect; L-NMMA decreased the effect	[4]
Study on pig pial arterioles using cranial windows	Topical application of 10^−6^–2 × 10^−4^ H_2_S with either 1–7 mM L-cysteine or glibenclamide	H_2_S and L-cysteine dilated the arterioles in a concentration-dependent manner, glibenclamide blocked the effect- L-cysteine increased the H_2_S level in the CSF	[49]
Examination of tissue samples after middle cerebral artery occlusion in Wistar rats	Effects of L-cysteine (2.5-5-10 mmol/kg ip. or 0.25-1.0-2.5 through intracerebroventricular injection) or 0.5 mmol/kg AOAA ip. on the outcome of stroke	L-cysteine dose-dependently increased the infarct volume by 30%; AOAA diminished the L-cys induced the increase of post-stroke area	[58]

**Table 4:** Ex vivo and in vivo investigations involving inhibitors of H_2_S producing enzymes.

Study Type	Experimental Situations	Major Conclusions	Ref
Ex vivo wire myograph study on middle cerebral artery segments from Sprague-Dawley rats	Effects of 0.1–10 mM L-cysteine alone and with 1 mM PAG or 1 mM/l AOAA on the vascular tone	PAG inhibited the L-cysteine induced vasorelaxation, but AOAA had no effect	[56]
Ex vivo wire myograph study on middle cerebral artery segments of WT and STZ-induced diabetic Sprague-Dawley rats	Examination of vascular tone after (10 µM–100 mM) L-cyteine and 20 mM PAG treatment	The effect of L-cysteine was attenuated by PAG both in the diabetic and control group	[52]
In vivo carotid ligation study on pial arteries of Wistar rats	Effects of 30 mM PAG on the Ach-induced vasodilation	PAG did not decrease Ach-induced vasodilation in the large vessels, just in the small pial arteries; After ischemia, the effect of PAG did not change significantly	[46]
In vivo study on meningeal arteries of Wistar rats using cranial windows	Effects of 2 mM oxamic acid (H_2_S inhibitor) on the NO mediated vasodilation	Oxamic acid reduced the blood flow increase evoked by the NO donor DEA NONOate	[4]
In vivo study on pig pial arterioles using cranial windows	Topical application of L-cysteine followed by 10 mM PAG or 1mM AOAA on the cranial windows	Hypercapnia increased the H_2_S level in the CSF given before but not during PAG treatment. AOAA had no effect.	[49]

**Table 5:** Ex vivo and in vivo studies involving knock-out animals and disease models.

Study Type	Experimental Situations	Major Conclusions	Ref
Ex vivo study on neonatal mouse (WT, HO2-null and CBS-null) vermis arterioles using cerebellar slices	Hypoxia	hypoxia elicited vasodilation, the effect was decreased both in HO-2-null and CBS-null mice	[48]
Ex vivo wire myograph study on basilar arteries of WT and CSE^−/−^ mice	Effects of RhoA agonist LPA and U46619 on vascular tone Effects of the Rho-associated protein kinase (ROCK) inhibitor Y27632 on CSE^−/−^mice	LPA and U46619 induced constriction, which was more pronounced in CSE^−/−^ mice; in CSE^−/−^ Y27632-induced relaxation was decreased	[53]
In vivo examination of mouse (WT, HO2-null and CBS-null) cortical precapillary arterioles by two-photon intravital laser scanning microscopy	Hypoxia with 10% O_2_	Hypoxia elicited vasodilation, which was decreased in HO-2-null and attenuated in CBS-null mice	[48]
In vivo study on pig pial arterioles using cranial windows	Hypercapnia with 10% CO_2_ for 5 min	H_2_S concentration in the CSF increased	[49]
In vivo study on pig pial arterioles using cranial windows	Effect of low (10^−12^–10^−11^) or high dose (10^−8^) endothelin-1, given with glibenclamide (K_ATP_ inhibitor) and paxilline (BK Ca inhibitor)	Low level of ET-1 induced vasodilation, which was attenuated after glibenclamide and paxilline. High level of ET-1 induced vasoconstriction, unaffected by glibenclamide or paxilline	[60]
In vivo human study on post-stroke patients	Examination of the linkage between serum cysteine levels and the outcome of stroke	Positive correlation between increased serum cysteine levels and poor clinical outcome	[58]

## Supplementary Materials

The following is available online at https://www.mdpi.com/2218-273X/10/12/1685/s1, Table S1: Summary of investigations into the role of H_2_S in cerebrovascular circulation.
